# Chronic stress, work-related daily challenges and medicolegal investigations: a cross-sectional study among German general practitioners

**DOI:** 10.1186/s12875-019-1032-6

**Published:** 2019-10-24

**Authors:** Christine Kersting, Lena Zimmer, Anika Thielmann, Birgitta Weltermann

**Affiliations:** 1 Institute for General Medicine, University Hospital Essen, University of Duisburg-Essen, Essen, Hufelandstraße 55, 45147 Essen, Germany; 2 Institute of General Practice and Family Medicine, University of Bonn, Sigmund-Freud-Straße 25, 53127 Bonn, Germany

**Keywords:** General practice, General practitioner, Chronic stress, Influencing factors, Medicolegal investigations, Challenges

## Abstract

**Background:**

The prevalence of chronic stress among German general practitioners (GPs) was shown to be twice as high as in the general population. Because chronic stress negatively influences well-being and poor physician well-being is associated with poor patient outcomes, targeted strategies are needed. This analysis focuses on work-related factors associated with high chronic stress in GPs.

**Methods:**

This cross-sectional study measured chronic stress among German GPs using the validated and standardized Trier Inventory for the Assessment of Chronic Stress (TICS-SSCS). Based on the TICS, GPs were categorized as either having low strain (≤ 25th percentile) or high strain (≥ 75th percentile) due to chronic stress. Questions on work-related challenges assessed the frequency and the subjectively perceived strain of single challenges. For exploratory analyses, these items were combined to dichotomous variables reflecting challenges that are common and that cause high strain. Variables significant in bivariate analyses were included in a multivariate logistic regression model analyzing their association with high chronic stress.

**Results:**

Data of 109 GPs categorized as having low strain (*n* = 53) or high strain (*n* = 56) due to chronic stress were analyzed. Based on bivariate analyses, challenges regarding personnel matters, practice software, complexity of patients, difficult patients, care facilities, scheduling of appointments, keeping medical records up-to-date, fee structures, and expectations versus reality of care were included in the regression model. Keeping medical records up-to-date had the strongest association with high chronic stress (odds ratio 4.95, 95% confidence interval 1.29–19.06). A non-significant trend showed that medicolegal investigations were more common among GPs with high chronic stress.

**Conclusions:**

This exploratory research shows that chronic stress is predominantly associated with administrative challenges. Treatment documentation, which represents a legal safeguard and is closely linked to existential concerns, has the strongest influence.

## Background

Chronic stress describes a feeling of being chronically overwhelmed due to an imbalance between the intensity or frequency of stressors and individual resources or compensation strategies during a prolonged period of time [1]. A number of studies have addressed strain due to chronic stress among workers in the health care sector. The majority of studies, however, focused on stress among hospital employees rather than physicians in the outpatient setting, especially general practitioners (GPs) [2–4].

Based on the validated and standardized Trier Inventory for the Assessment of Chronic Stress (TICS-SSCS), a previous analysis of this cross-sectional study showed a prevalence of high strain due to chronic stress that was twice as high in German GPs compared to the German general population [1, 5].

Since chronic stress is associated with an increased risk for poor well-being, and poor physician well-being was shown to be associated with poor patient outcomes and a higher incidence of medical errors [6], approaches addressing chronic stress reduction among GPs might help improve patient care. In order to provide targeted approaches, factors associated with chronic stress need to be identified. The influence of practice, physician, and employment-related characteristics was already assessed in our previous analysis: Working ≥60 h per week increases the probability of high chronic stress, while applying more than five stress-compensating measures regularly was identified as a preventive factor [5]. However, chronic stress was not associated with GPs’ demographic characteristics, which is consistent with a study among Canadian GPs [5, 7]. Beside such individual characteristics and work-related conditions, GPs face different occupational and health care system-related challenges in everyday practice. In this context, studies among Canadian GPs conducted by Lee et al. (2008, 2009) showed that the amount of paperwork, high workload, scarce time, long waiting lists to see specialists, diagnostic tests, challenges concerning documentation and practice management, community resources, imposed regulations, feeling undervalued, financial concerns, and challenging patients are key stressors [7, 8].

Indeed, these findings from Canada might not be representative for and transferable to German GPs due to the differences in the health care systems. Classically, German GP practices are solo practices with self-employed physicians. Thus, GP practices are small- and medium-sized enterprises (SME) [9]. As a result, challenges of daily work are not limited to aspects concerning medical activities and patient care, but also include administrative and entrepreneurial tasks as well as existential responsibility. Aiming to provide further insights into the influence of such diverse challenges on strain due to chronic stress, this exploratory research assesses factors associated with high chronic stress according to the TICS-SSCS in comparison to low chronic stress by focusing on issues relating to practice equipment, personnel, patients, practice organization, cooperation with external colleagues or health care providers, being a doctor, medicolegal investigations, and allegations as challenges that shape the daily medical practice of German GPs.

## Methods

### Study design and study population

The initial study was conducted as a cross-sectional survey among GPs and practice assistants working in the 185 general practices of the practice network of the Institute for General Medicine, University Hospital Essen, Germany. The practices affiliated with the network are located in urban and rural regions of North-Rhine-Westphalia (Western Germany) at an average distance of 30 km to the university hospital. All practices were invited to participate. Data was collected during on-site visits between April 2014 and September 2014 using a self-administered questionnaire. Details on the recruitment strategy and the data collection method are published elsewhere [5].

All participants received written information and signed informed consent forms. Ethical approval was obtained from the Ethics Committee of the Medical Faculty of the University of Duisburg-Essen (reference number: 13–5536-BO, date of approval: 11/24/2014).

### Study instruments

Practice characteristics (e.g., practice type, number of team members differentiated by professional groups, number of patients per quarter) were available for every practice as all practices complete a questionnaire on practice characteristics when joining the network and provide informed consent on using these data within the scope of research projects.

Participant characteristics were assessed by questions addressing socio-demographic characteristics, including age, sex or marital status and work-related characteristics, e.g., working status, working hours and work satisfaction.

Chronic stress was measured using the short version of the standardized and validated TICS-SSCS [10, 11]. This psychometric questionnaire comprises 12 items which assess strain due to chronic stress three months retrospectively on a 5-point Likert scale ranging from 0 (never) to 4 (very often) [10]. The reliability of the chronic stress screening scale accounts for Cronbach’s α = 0.91 [11]. The TICS was chosen as it allows for a comparison of chronic stress in our study population with the general population reported in the ‘German Health Interview and Examination Survey for Adults’ (DEGS1) [1, 5]. An English translation of the TICS-SSCS is provided in the Additional file 1: Table S1.

Daily work-related challenges were addressed by questions that assessed the frequency of diverse challenges in the last three months (daily, weekly, monthly, seldom, never) and the strain associated with these challenges on a 5-point Likert scale ranging from 0 (none) to 4 (very high). The challenges were divided into the categories techniques/practice equipment (6 items), personnel issues (11 items), patient issues (10 items), practice processes/organization (7 items), cooperation with external medical colleagues (7 items), cooperation with other health care providers (4 items), and being a doctor (5 items). In addition, 12 items addressing the frequency of medicolegal investigations and three items addressing the frequency of allegations of treatment errors since being a GP (0 to ≥4 times) as well as the associated strain (5-point Likert scale ranging from none to very high) were assessed. The challenges addressed in the questionnaire were selected on the basis of an informal discussion with GPs regarding typical work contents. For a comprehensive list of challenges see Additional file 1: Table S2.

### Statistical analysis

This exploratory analysis comprised data from all GPs who provided their answers to the TICS-SSCS. The TICS-SSCS was analyzed by adding all values to a sum-score which reflects subjective strain due to chronic stress ranging from 0 (never stressed) to a maximum of 48 (very often stressed). Based on each GP’s sum-score, participants were categorized as having low, medium or high strain due to chronic stress using the gender-specific ≤25th and the ≥75th percentile of the respective distribution of the study population. For male GPs the cut-off points were ≤ 9 for low, 10–19 for medium and ≥ 20 for high strain; for female GPs the cut-off points were ≤ 13, 14–24, and ≥ 25, respectively. We used gender-specific cut-offs as other studies have shown that age, gender, and socio-economic status influenced the reporting behavior of the TICS-SSCS items [1, 11]. Only those GPs who were categorized as having low strain or high strain due to chronic stress were included in the analyses. A power calculation was conducted to analyze whether the participants included in the analyses were appropriate to detect differences between these two groups.

The frequency and strain of the various work-related challenges were combined to dichotomous variables as follows:

- Challenges of daily work were combined to dichotomous variables representing challenges which occur daily or weekly and are associated with high or very high strain.
- For medicolegal investigations, variables were combined to dichotomous variables representing challenges which had occurred ≥4 times since being a doctor and were associated with high or very high strain.
- For allegations of treatment errors, variables were combined to dichotomous variables representing challenges which had occurred at least once since being a doctor and were associated with high or very high strain.

In order to identify factors associated with high strain due to chronic stress, multivariate logistic regression models were performed. The first model included all GPs, while the second included self-employed GPs only. Independent determinants were the dichotomous variables on common, straining challenges. A test for collinearity was carried out and only those determinants significant in previous Chi-square tests (Fisher’s exact test if cells were < 5) were included in the regression analysis. The nominal significance level for these bivariate analyses was *p* < 0.05. To correct for multiple testing, the Benjamini-Hochberg procedure was applied, which controls the false discovery rate at the nominal *p*-value [12]. Factors associated with high strain are described as odds ratios (OR) with a 95% confidence interval (CI). Nagelkerke R2 is reported as a goodness-of-fit indicator for both logistic regression models. All statistical analyses were performed using IBM SPSS Statistics for Windows, Version 24 (Armonk, NY: IBM Corp.). Percentages and mean values are reported for valid cases.

## Results

### Study characteristics

226 GPs from 137 practices participated in the study (practice response rate: 74.1%). Data of 214 GPs from 129 practices were eligible for analysis. Of those GPs, 141 (65.9%) were male (Table 1). The mean age was 51.8 years (standard deviation (SD): 8.7). 158 GPs worked in group practices (74.9%), while 185 were self-employed (87.3%). Two-thirds of the practices were group practices (75 of 129, 59.5%).

**Table 1:** Characteristics of the participating general practitioners, stratified by low and high strain due to chronic stress

	All GPs (*n* = 214)	GPs with low strain due to chronic stress (*n* = 53)	GPs with high strain due to chronic stress (*n* = 56)
Sex, *n* (%)			
Male	141 (65.9)	33 (62.3)	38 (67.9)
Female	73 (34.1)	20 (37.7)	18 (32.1)
Age, mean ± SD (range)	51.8 ± 8.7 (27–74)	52.1 ± 9.5 (27–71)	51.2 ± 9.0 (33–65)
Physician in GP training, *n* (%)			
Yes	7 (3.3)	2 (3.8)	2 (3.6)
No	207 (3.3)	51 (96.2)	54 (96.4)
Marital status, *n* (%)			
Single	16 (7.5)	5 (9.4)	4 (7.3)
Married	188 (88.7)	43 (81.1)	50 (90.9)
Divorced	8 (3.8)	5 (9.4)	1 (1.8)
Number of persons in household, *n* (%)			
≤ 3	145 (67.8)	38 (71.7)	41 (73.2)
≥ 4	69 (32.2)	15 (28.3)	15 (26.8)
Employment status, *n* (%)			
Self-employed	185 (87.3)	44 (86.3)	48 (85.7)
Employed	27 (12.7)	7 (13.7)	8 (14.3)
Employment level, *n* (%)			
Full-time	190 (90.5)	45 (88.2)	52 (94.5)
Part-time	20 (9.5)	6 (11.8)	3 (5.5)
Working hours per week, *n* (%)			
≤39 h	52 (24.4)	20 (38.5)	10 (17.9)
40–59 h	116 (54.5)	22 (42.3)	25 (44.6)
≥ 60 h	45 (21.1)	10 (19.2)	21 (37.5)
Practice type, *n* (%)			
Solo practice	53 (25.1)	17 (32.7)	16 (29.1)
Group practice	158 (74.9)	35 (67.3)	39 (70.9)
Work experience, mean ± SD (range)			
Years in job	23.0 ± 9.2 (1–44)	23.2 ± 10.2 (1–44)	22.8 ± 9.2 (4–37)
Years in current practice	14.3 ± 9.4 (0–37)	15.9 ± 10.7 (0–37)	14.3 ± 9.0 (0–32)

Percentages and mean values are reported for valid cases

Of the 214 GPs, only those categorized as having low strain due to chronic stress (*n* = 53) or as having high strain due to chronic stress (*n* = 56) were included in the analyses. The power calculation revealed that this sample size (*n* = 109) was sufficient to achieve 80% power to detect a 10% difference between GPs with low strain and high strain due to chronic stress with a significance level of 0.05 (two-sided). In case of greater differences, the power achieved was even higher (e. g., 12% difference: 90% power; 14% difference: 95% power).

The 109 GPs worked in 61 different practices, 27 of which were group practices (45.8%). 71 GPs were male (65.1%), their mean age was 51.6 (SD: 9.2). 74 GPs worked in group practices (69.2%) and 92 were self-employed (86.0%). Characteristics of GPs with low and high strain did not differ, except that those with higher stress more frequently worked ≥60 h per week than those with lower stress (37.5% versus 19.2%, *p* = 0.026). More information on socio-demographic and work-related characteristics is provided in Table 1.

### Comparison of participants with low and high strain due to chronic stress

**Fig. 1:**
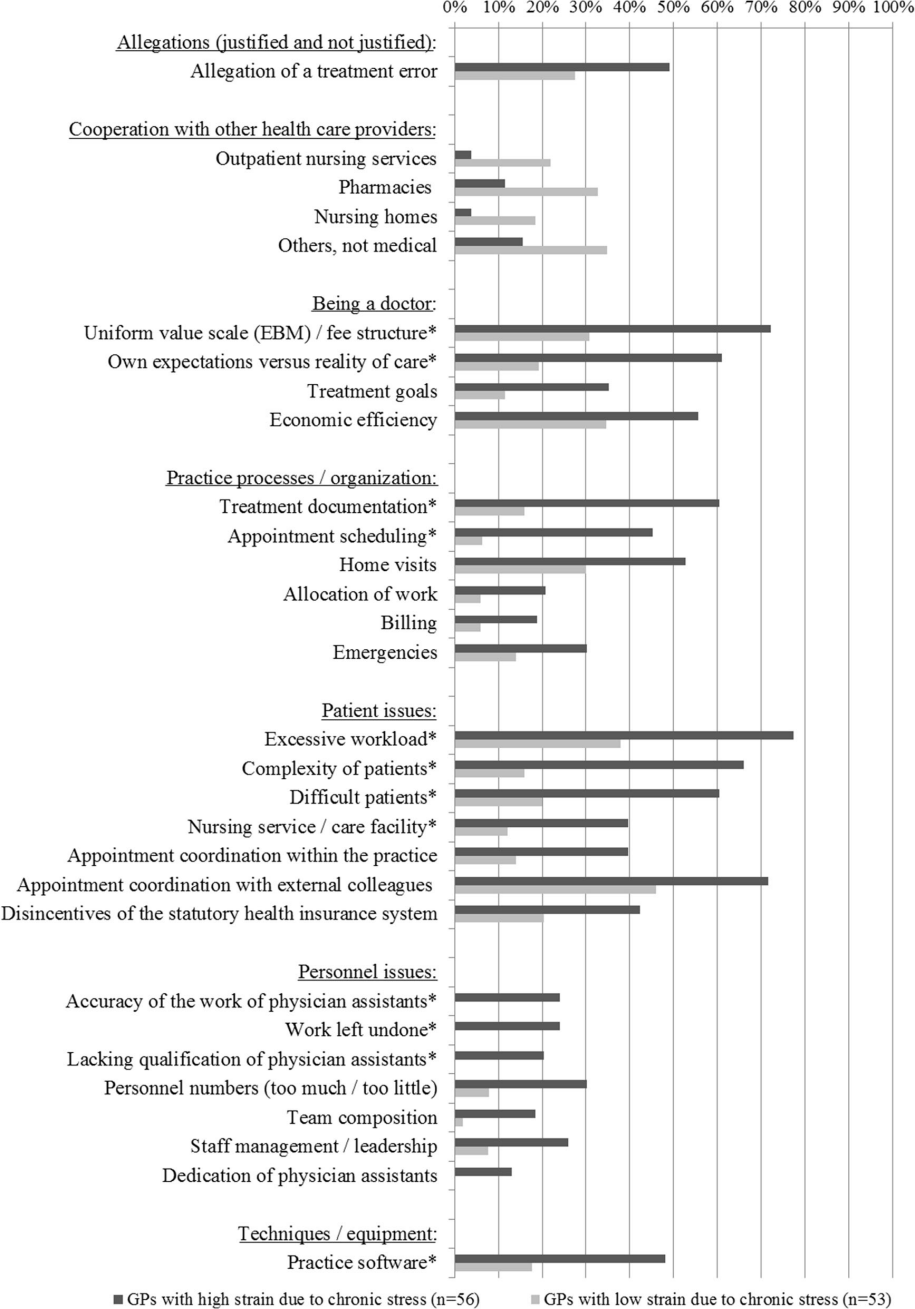
Common, straining challenges of daily practice stratified by general practitioners with low and high strain due to chronic stress; *Significant after correcting for multiple testing (*p*-values see Additional file 1: Table S3)

Comparing GPs with low and high stress levels showed that those with high strain due to chronic stress more frequently experienced challenges regarding personnel issues, patient issues, practice organization, the role of being a doctor, cooperation with other health care providers, practice software, and allegations of treatment errors (Fig. 1). After correcting for multiple testing according to Benjamini Hochberg, differences at a significance level of *p* ≤ 0.001 remained significant (Additional file 1: Table S3). Determinants that remained significant after correcting for multiple testing were included in the multivariate logistic regression model.

### Factors associated with high strain due to chronic stress

Due to missing values for the independent variables, data of nine GPs were omitted in the multivariate regression analysis. Based on data of *n* = 100 GPs, the analysis showed that challenges of keeping medical records up-do-date had the strongest association with high strain due to chronic stress (OR 4.95, 95% CI: 1.29–19.06), followed by conflicts between own expectations and the reality of care, and practice management (OR 4.22, 95% CI 1.00–17.77, not significant), nursing services/care facilities in the event of patient issues (OR 3.56, 95% CI 0.67–18.87, not significant), and practice software issues (OR 3.33, 95% CI 0.79–14.00, not significant) (Table 2). The Nagelkerke R2 (0.721) indicates that this model accounts for 72.1% of the variability in the dependent variable high strain due to chronic stress.

**Table 2:** Logistic regression model for all GPs (*n* = 100): Association between frequent, straining challenges of daily general practice and high strain due to chronic stress (dependent variable)

Commonly (high) strained due to …	Odds Ratio	95% confidence interval	Beta	Standard error	*p*-value
Keeping medical records up-to-date	4.95	1.29–19.06	1.60	0.69	0.020
Conflicts between own expectations and the reality of care	4.22	1.00–17.77	1.44	0.73	0.050
Appointment scheduling	0.23	0.04–1.17	−1.48	0.83	0.076
Practice software issues	3.33	0.79–14.00	1.20	0.73	0.101
Nursing services/care facilities in the event of patient issues	3.56	0.67–18.87	1.27	0.85	0.136
EBM / fee structure	2.49	0.61–10.14	0.91	0.72	0.202
Communication with difficult patients	2.44	0.55–10.93	0.89	0.77	0.243
Complexity of patients	2.04	0.39–10.57	0.71	0.84	0.396
Excessive caseload	1.61	0.33–7.95	0.48	0.81	0.558
Work left undone*	–	–	–	–	–
Accuracy of work of physician assistants*	–	–	–	–	–
Lacking qualification of physician assistants*	–	–	–	–	–

*no reasonable estimates effectively predicting the dependent variable available due to quasi-selection (see distribution of the variables in Fig. 1)

Focusing on self-employed GPs (including 86 of 92 GPs), the results of the multivariate regression analysis did not change (Table 3): Only challenges due to keeping medical records up-to-date were significantly and strongly associated with high strain due to chronic stress: OR 4.38, 95% CI: 1.10–17.56. The Nagelkerke R2 (0.683) indicates that this model accounts for 68.3% of the variability in the dependent variable.

**Table 3:** Logistic regression model for self-employed GPs (*n* = 92): Association between frequent, straining challenges of daily general practice and high strain due to chronic stress (dependent variable)

Commonly (high) strained due to …	Odds Ratio	95% confidence interval	Beta	Standard error	*p*-value
Keeping medical records up-to-date	4.38	1.10–17.56	1.48	0.71	0.037
Conflicts between own expectations and the reality of care	3.88	0.92–16.38	1.36	0.74	0.065
Appointment scheduling	0.31	0.06–1.59	−1.18	0.84	0.159
Nursing services/care facilities in the event of patient issues	3.12	0.61–16.60	1.16	0.84	0.168
EBM / fee structure	2.58	0.61–10.87	0.95	0.73	0.197
Communication with difficult patients	2.43	0.53–11.23	0.89	0.78	0.255
Practice software issues	2.31	0.52–10.17	0.84	0.76	0.270
Complexity of patients	2.31	0.41–12.86	0.84	0.88	0.341
Excessive caseload	1.18	0.22–6.50	0.17	0.87	0.847
Work left undone*	–	–	–	–	–
Accuracy of work of physician assistants*	–	–	–	–	–
Lacking qualification of physician assistants*	–	–	–	–	–

*no reasonable estimates effectively predicting the dependent variable available due to quasi-selection (see distribution of the variables in Fig. 1)

## Discussion

Our exploratory analyses shows that foreseeable dimensions of patient care, such as a high caseload or difficult patients, but mostly practice management-related administrative and entrepreneurial challenges, are associated with high stress among German GPs: personnel matters, appointment scheduling, conflicts between own expectations and the reality of care, practice management, issues related to the fee structure, software problems, and keeping medical records up-to-date. Although GPs reported that medicolegal investigations cause high subjective strain, these challenges did not correlate with high stress levels measured by TICS. This is feasible, since TICS measures chronic stress in the last three months and medicolegal investigations are relatively rare.

Despite differences between the German and the Canadian (primary care) health care systems, our results are in line with studies among Canadian family physicians which show that patient-, practice management- and health care system-related challenges are key stressors [7, 8]. The fact that most of the stressors identified in our study are organizational challenges of SME and being self-employed fits the composition of the study sample which included about 90% self-employed practice owners. The results demonstrate that the autonomy associated with being self-employed is also burdensome. Autonomy as a ‘double-edged sword’ [13] is not typical for GPs at all, but was described among self-employed workers across businesses: Although self-employment implies a higher job autonomy, which is associated with greater control over the organization and processes, this autonomy is described as superficial, since owners of SME must adhere to imposed regulations from the respective market or system and have greater jobs demands, responsibility pressure, and existential concerns [13–15]. Focusing on physicians, Wallace et al. (2009) describe a decline in autonomy due to increasing external control, e.g. by the health care system or patients [6].

Addressing existential concerns, Lewin-Epstein and Yuchtman-Yaar (1991) emphasize that, compared to employed workers, not only job and income is at stake for self-employed persons, but also property, i.e. their existence, which means that self-employed persons are aware that their actions and decisions have a direct impact on their enterprise and work setting [13]. This might explain the result of the regression model: According to our data, GPs who reported being (highly) strained due to issues relating to daily or weekly treatment documentation were nearly five times more likely to suffer from high chronic stress. The burden related to documentation tasks depends on the caseload: German GPs have average consultation times of seven to eight minutes per patient and are responsible for 800 patients per quarter, or even as many as 1800 patients in larger practices. However, when considering treatment documentation from the perspective of German GPs, accurate documentation is not only part of good patient care, but also has legal implications with regard to liability and serving as evidence [16]. Due to the special circumstance that German GP practices are typically SME with self-employed practice owners, keeping medical records up-to-date is a very important aspect of their work which is closely linked to their existence. Nevertheless, this result is difficult to compare to the literature as the relationship between existential concerns and chronic stress among GPs has not been described in other studies yet. Only Lee et al. (2009) described financial concerns as a key stressor among Canadian family physicians based on only ten explorative in-depth interviews [8]. However, the association between existential concerns or anxiety about the future and distress is well-documented in other samples and settings; for example, among chronically ill patients, including young- and middle-aged patients with cancer or adults with diabetes [17, 18], medical students considering dropping out of their medical training program [19], or, as already mentioned above, self-employed workers across sectors [13, 15].

### Strength and limitations

A core strength of our study is the high response rate which might indicate a strong interest in the topic; however, this may also be explained by the on-site visits conducted for data collection [5] and the practices’ affiliation with the institute. Although all participating practices were affiliated with the same institute, a selection bias can be excluded as the institutes’ practice sample was shown to be representative for German GP practices [20].

Despite sufficient power to detect group differences in the small sample analyzed, the results of the logistic regression models are limited as the analyses were exploratory and included multiple testing. Multiple testing was addressed by applying the Benjamini-Hochberg procedures, but this approach is less stringent compared to more conservative approaches like Bonferroni. Moreover, it cannot be excluded that the wide variety of challenges assessed by the questionnaire was not exhaustive. However, the effect of this bias is estimated to be low, since GPs had were given the opportunity to add straining challenges as free text, which was barely used. Due to the cross-sectional design our results merely indicate associations.

## Conclusions

German GP practices are SME. Due to this, they are confronted not only with difficulties related to patient care, but also with a variety of entrepreneurial and administrative challenges. Especially the workload needed to keep medical records up-to-date was shown to be associated with high strain due to chronic stress. As this mandatory documentation represents a legal safeguard, it is closely linked to financial and even existential concerns, which have been described as stressors in the literature. Yet, such entrepreneurial and administrative tasks are necessary and inevitable in the daily practice of a SME. Considering that each practice has its own procedures for handling such challenges, targeted approaches which empower practices to organize their practice administration and manage the SME are needed.

## Supplementary information

**Additional file 1: Table S1.** Non-validated English translation of the 12 TICS-SSCS items, which assess straining experiences in the last three months on a 5-point Likert scale ranging from 0 (never) to 4 (very often). **Table S2.** General practice challenges assessed in the cross-sectional study. **Table S3.** Correction for multiple testing according to Benjamini Hochberg.

## Data Availability

The dataset used and/or analyzed during the study is available from the corresponding author on reasonable request.

## Abbreviations

CI: Confidence interval
EBM: Uniform value scale
GP: General practitioner
OR: Odds Ratio
SD: Standard deviation
SME: Small- and medium-sized enterprise
TICS-SSCS: Trier Inventory for the Assessment of Chronic Stress

## References

[1] Hapke U, Maske UE, Scheidt-Nave C, Bode L, Schlack R, Busch MA (2013). Chronic stress among adults in Germany: results of the German health interview and examination survey for adults (DEGS1). Bundesgesundheitsblatt Gesundheitsforschung Gesundheitsschutz.

[2] Bernburg M, Vitzthum K, Groneberg DA, Mache S (2016). Physicians' occupational stress, depressive symptoms and work ability in relation to their working environment: a cross-sectional study of differences among medical residents with various specialties working in German hospitals. BMJ Open.

[3] Bauer J, Groneberg DA (2013). Distress among physicians in hospitals – an investigation in Baden-Württemberg, Germany. Dtsch Med Wochenschr.

[4] Khamisa N, Peltzer K, Ilic D, Oldenburg B (2016). Work related stress, burnout, job satisfaction and general health of nurses: a follow-up study. Int J Nurs Pract.

[5] Viehmann A, Kersting C, Thielmann A, Weltermann B (2017). Prevalence of chronic stress in general practitioners and practice assistants: personal, practice and regional characteristics. PLoS One.

[6] Wallace JE, Lemaire JB, Ghali WA (2009). Physician wellness: a missing quality indicator. Lancet..

[7] Lee FJ, Stewart M, Brown JB (2008). Stress, burnout, and strategies for reducing them: what's the situation among Canadian family physicians?. Can Fam Physician.

[8] Lee FJ, Brown JB, Stewart M (2009). Exploring family physician stress: helpful strategies. Can Fam Physician.

[9] Reed M, Lehmann B, Herrmann M (2017). The envolving state of general practice and GP education in Germany. Health Care Current Reviews.

[10] Schulz P, Schlotz W, Becker P (2004). TICS. Trierer Inventar zum chronischen Stress.

[11] Petrowski K, Paul S, Albani C, Brahler E (2012). Factor structure and psychometric properties of the trier inventory for chronic stress (TICS) in a representative German sample. BMC Med Res Methodol.

[12] McDonald JH. Handbook of biological statistics. 3rd ed. Baltimore, Maryland: Sparky House Publishing; 2014.

[13] Lewin-Epstein N, Yuchtman-Yaar E (1991). Health risks of self-employment. Work Occup.

[14] Buttner EH (1992). Entrepreneurial stress: is it hazardous to your health?. J Manag Issues.

[15] Prottas DJ, Thompson CA (2006). Stress, satisfaction, and the work-family interface: a comparison of self-employed business owners, independents, and organizational employees. J Occup Health Psychol.

[16] Schirmer HD, Hübner M (2009). Ärztliche Dokumentationspflichten: Das Ende der Fahnenstange. Deutsches Ärzteblatt.

[17] Meeker CR, Wong Y-N, Egleston BL, Hall MJ, Plimack ER, Martin LP (2017). Distress and financial distress in adults with Cancer: an age-based analysis. J Natl Compr Cancer Netw.

[18] Dennick K, Sturt J, Speight J (2017). What is diabetes distress and how can we measure it? A narrative review and conceptual model. J Diabetes Complicat.

[19] McLuckie A, Matheson KM, Landers AL, Landine J, Novick J, Barrett T, Dimitropoulos G (2018). The relationship between psychological distress and perception of emotional support in medical students and residents and implications for educational institutions. Acad Psychiatry.

[20] Viehmann A, Thielmann A, Gesenhues S, Weltermann BM (2014). Do academic family practices reflect routine primary care?: a methodological approach. Z Allg Med.

