# Supplementary material for: Chronic stress, work-related daily challenges and medicolegal investigations: a cross-sectional study among German general practitioners
Source: BMC Fam Pract. 2019 Oct 24;20:143. doi: 10.1186/s12875-019-1032-6 (PMC6813962; doi:10.1186/s12875-019-1032-6)
Supplement: Supplementary file 1 — Additional file 1: Table S1. Non-validated English translation of the 12 TICS-SSCS items, which assess straining experiences in the last three months on a 5-point Likert scale ranging from 0 (never) to 4 (very often). Table S2. General practice challenges assessed in the cross-sectional study. Table S3. Correction for multiple testing according to Benjamini Hochberg. [file 12875_2019_1032_MOESM1_ESM.docx]

**Appendix**

| **Table 1. Non-validated English translation of the 12 TICS-SSCS items, which** **assess straining experiences in the last three months on a 5-point Likert scale ranging from 0 (never) to 4 (very often)** | |
| --- | --- |
| **German items** | **English translation** |
| Befürchtung, dass irgendetwas Unangenehmes passiert | I feared that something unpleasant might happen |
| Ich bemühe mich vergeblich, mit guten Leistungen Anerkennung zu erhalten | I tried, to no avail, to get appreciation through excellent performance |
| Zeiten, in denen ich zu viele Verpflichtungen zu erfüllen habe | There were times I had too many obligations |
| Zeiten, in denen ich sorgenvolle Gedanken nicht unterdrücken kann | There were times I could not suppress my worries |
| Obwohl ich mein bestes gebe, wird meine Arbeit nicht gewürdigt | Although I did my best, my work was not valued |
| Erfahrung, dass alles zu viel ist, was ich zu tun habe | I felt that everything I had to deal with became too much |
| Zeiten, in denen ich mir viele Sorgen mache und nicht damit aufhören kann | There were times I could not stop worrying |
| Zeiten, in denen ich nicht die Leistung bringe, die von mir erwartet wird | There were times I was unable to meet others’ expectations |
| Zeiten, in denen mir die Verantwortung für andere zur Last wird | There were times I felt burdened by the responsibility for others |
| Zeiten, in denen mir die Arbeit über den Kopf wächst | There were times I felt overwhelmed with work |
| Befürchtung, meine Aufgaben nicht erfüllen zu können | I worried that I would not be able to accomplish my work |
| Zeiten, in denen mir die Sorgen über den Kopf wachsen | There were times I felt overwhelmed by worries |

| **Table 2. General practice challenges assessed in the cross-sectional study** | | |
| --- | --- | --- |
| **Category** | **Questions** | **Challenges addressed in this category / question** |
| Techniques / practice | In the last 3 months: How often were you occupied with the following challenges regarding techniques / practice equipment?  To what extent did you feel stressed due to these challenges? | - Office technology - Software - Floor plan - Rental issues - Defects (heating / sanitary / mold) - Medical equipment |
| Personnel issues | In the last 3 months: How often were you occupied with the following challenges regarding personnel issues?  To what extent did you feel stressed due to these challenges? | - Personnel numbers (too much / too little) - Sick leave - Work schedule - Workplace organization / conditions - Staff management / leadership - Lacking qualification of practice assistants - Accuracy of the work of practice assistants - Work left undone - Costs - Dedication of physician assistants - Team composition |
| Patient issues | In the last 3 months: How often were you occupied with the following challenges regarding patient issues?  To what did you feel stressed due to these challenges? | - Excessive caseload - Complexity of patients - Appointment coordination within the practice - Appointment coordination with external colleagues - Disease management program - Regulations after hospital discharge - Nursing service / care facility - Difficult patients (communication) - Best choice treatment versus services provided by statutory health insurance - Disincentives of the statutory health insurance system |
| Practice processes / organization | In the last 3 months: How often were you occupied with the following challenges regarding practice processes / organization?  To what extent did you feel stressed due to these challenges? | - Appointment scheduling - Allocation of work - Orders / inventory control - Billing - Keeping medical records up-to-date - Home visits - Emergencies |
| Cooperation with external medical colleagues | In the last 3 months: How often were you occupied with the following challenges regarding cooperation with external medical colleagues?  To what extent did you feel stressed due to these challenges? | - Agreements with colleagues - Expertise / professional competence of colleagues - Treatment strategy of colleagues - Diagnosis made by colleagues - Appointment coordination with colleagues - Prescribing behavior of colleagues - Treatment errors of colleagues |
| Cooperation with other health care providers | Within the last 3 months: How often were you occupied with the following challenges regarding cooperation with other health care providers?  To what extent did you feel stressed due to these challenges? | - Pharmacies - Outpatient nursing services - Nursing homes - Others, not medical |
| Being a doctor | Within the last 3 months: How often were you occupied with the following challenges regarding being a doctor?  To what extent did you feel stressed due to these challenges? | - Acceptance as doctor among patients - Treatment goals - Economic efficiency - Uniform values scale (EBM) / fee structure - Conflicts between own expectations versus reality of care |
| Medicolegal investigations | Within the last 3 months: How often were you occupied with the following challenges regarding medicolegal investigations?  To what extent did you feel stressed due to these challenges? | - Efficiency audit / performance audit - Narcotic drug examination - Pharmaceutical examination - Remedy examination - Plausibility check - Mandatory advanced education not fulfilled - Fire protection inspection - Sanitation inspection (health department) - Professional association proceedings - Enforcement of penal proceedings - Civil-law proceedings with colleagues - Problems with the admission committee |
| Allegations (justified and not justified) | Within the last 3 months: How often were you occupied with the following challenges regarding allegations (justified and not justified)?  To what extent did you feel stressed due to these challenges? | - Allegation of a treatment error - Review procedure to own liability case - Judicial proceedings to own liability case |

| **Table 3. Correction for multiple testing according to Benjamini Hochberg** | | | | | |
| --- | --- | --- | --- | --- | --- |
| Combination variables:  Commonly (high) strained due to… | GPs with low strain due to chronic stress (n=53),  n (%) | GPs with high strain due to chronic stress  (n=56),  n (%) | p-value | Rank | (i/m)Q* |
| Personnel issues: Accuracy of the work of physician assistants | 0 (0)0 | 13 (24.1) | 0.000 | 1 | 0.00077 |
| Personnel issues: Work left undone | 0 (0)0 | 13 (24.1) | 0.000 | 1 | 0.00077 |
| Practice processes/ practice organization: Keeping medical records up-to-date | 8 (16.0) | 32 (60.4) | 0.000 | 1 | 0.00077 |
| Practice processes/ practice organization: Appointment scheduling | 3 (6.4) | 24 (45.3) | 0.000 | 1 | 0.00077 |
| Patient issues: Excessive workload | 19 (38.0) | 41 (77.4) | 0.000 | 1 | 0.00077 |
| Patient issues: Complexity of patients | 8 (16.0) | 35 (66.0) | 0.000 | 1 | 0.00077 |
| Patient issues: Difficult patients (communication) | 10 (20.0) | 32 (60.4) | 0.000 | 1 | 0.00077 |
| Being a doctor: Uniform value scale (EBM) / fee structure | 16 (30.8) | 39 (72.2) | 0.000 | 1 | 0.00077 |
| Being a doctor: Conflict between own expectations and reality of care | 10 (19.2) | 33 (61.1 | 0.000 | 1 | 0.00077 |
| Techniques / equipment: Software | 9 (17.6) | 26 (48.1) | 0.001 | 2 | 0.0015 |
| Personnel issues: Lacking qualification of physician assistants | 0 (0) | 11 (20.4) | 0.001 | 2 | 0.0015 |
| Patient issues: Nursing service/care facility | 6 (12.0) | 21 (39.6) | 0.001 | 2 | 0.0015** |
| Patient issues: Appointment coordination within the practice | 7 (14.0) | 21 (39.6) | 0.003 | 3 | 0.0023 |
| Personnel issues: Personnel numbers (too much / too little) | 4 (7.8) | 16 (30.2) | 0.004 | 4 | 0.0031 |
| Being a doctor: Treatment goals | 6 (11.5) | 19 (35.2) | 0.004 | 4 | 0.0031 |
| Personnel issues: Team composition | 1 (1.9) | 10 (18.5) | 0.005 | 5 | … |
| Cooperation with other health care providers: Outpatient nursing services | 11 (22.0) | 2 (3.8) | 0.006 | 6 |  |
| Patient issues: Appointment coordination with external colleagues | 23 (46.0) | 38 (71.7) | 0.008 | 7 |  |
| Cooperation with other health care providers: Pharmacies | 16 (32.7) | 6 (11.5) | 0.010 | 8 |  |
| Personnel issues: Staff management / leadership | 4 (7.7) | 14 (25.9) | 0.012 | 9 |  |
| Personnel issues: Dedication of physician assistants | 0 (0) | 7 (13.0) | 0.013 | 10 |  |
| Patient issues: Disincentives of the statutory health insurance system | 10 (20.4) | 22 (42.3) | 0.018 | 11 |  |
| Cooperation with other health care providers: Nursing homes | 9 (18.4) | 2 (3.8) | 0.019 | 12 |  |
| Practice processes / practice organization: Home visits | 5 (30.0) | 28 (52.8) | 0.019 | 12 |  |
| Allegations (justified and not justified): Allegation of a treatment error | 14 (27.5) | 27 (49.1) | 0.022 | 13 |  |
| Practice processes / practice organization: Allocation of work | 3 (6.0) | 11 (20.8) | 0.029 | 14 |  |
| Being a doctor: Economic efficiency | 18 (34.6) | 30 (55.6) | 0.030 | 15 |  |
| Cooperation with other health care providers: Others, not medical | 16 (34.8) | 7 (15.6) | 0.035 | 16 |  |
| Practice processes / practice organization: Billing | 3 (6.0) | 10 (18.9) | 0.049 | 17 |  |
| Practice processes / practice organization: Emergencies | 7 (14.0) | 16 (30.2) | 0.049 | 17 |  |
| Techniques / equipment: Floor plan | 0 (0) | 5 (9.3) | 0.057 | 18 |  |
| Techniques / equipment: Rental issues | 0 (0) | 5 (9.3) | 0.057 | 18 |  |
| Personnel issues: Costs | 1 (1.9) | 7 (13.2) | 0.060 | 19 |  |
| Medicolegal investigation: Mandatory advanced education not fulfilled | 36 (81.8) | 33 (64.7) | 0.062 | 20 |  |
| Medicolegal investigation: Professional association proceedings | 38 (86.4) | 36 (70.6) | 0.065 | 21 |  |
| Patient issues: Best choice treatment versus services provided by statutory health insurance | 7 (14.0) | 15 (28.8) | 0.068 | 22 |  |
| Medicolegal investigation: Plausibility check | 29 (63.0) | 23 (46.9) | 0.115 | 23 |  |
| Techniques / equipment: Defects (heating/sanitary/mold) | 0 (0) | 4 (7.4) | 0.118 | 24 |  |
| Cooperation with external medical colleagues: Appointment coordination with colleagues | 16 (31.4) | 10 (18.9) | 0.141 | 25 |  |
| Patient issues: Regulations after hospital discharge | 15 (30.0) | 23 (43.4) | 0.159 | 26 |  |
| Allegations (justified and not justified): Judicial proceedings to own liability case | 2 (4.1) | 7 (13.0) | 0.165 | 27 |  |
| Allegations (justified and not justified): Review procedure to own liability case | 6 (12.2) | 12 (22.2) | 0.183 | 28 |  |
| Techniques / equipment: Medical equipment | 1 (2.0) | 5 (9.3) | 0.206 | 29 |  |
| Techniques / equipment: Office technology | 10 (19.6) | 16 (29.6) | 0.234 | 30 |  |
| Medicolegal investigation: Problems with the admission committee | 0 (0) | 3 (5.8) | 0.246 | 31 |  |
| Medicolegal investigation: Efficiency audit / performance audit | 20 (41.7) | 17 (31.5) | 0.286 | 32 |  |
| Being a doctor: Acceptance as doctor among patients | 3 (5.8) | 7 (13.0) | 0.320 | 33 |  |
| Medicolegal investigation: Remedy examination | 29 (61.7) | 27 (51.9) | 0.327 | 34 |  |
| Medicolegal investigation: Sanitation inspection (health department) | 30 (68.2) | 30 (58.8) | 0.346 | 35 |  |
| Cooperation with external medical colleagues: Prescribing behavior of colleagues | 9 (17.6) | 6 (11.5) | 0.380 | 36 |  |
| Medicolegal investigation: Pharmaceutical examination | 22 (47.8) | 21 (40.4) | 0.459 | 37 |  |
| Practice processes / practice organization: Orders / inventory control | 0 (0) | 2 (3.8) | 0.496 | 38 |  |
| Medicolegal investigation: Civil-law proceedings with colleagues | 0 (0) | 2 (3.8) | 0.498 | 39 |  |
| Patient issues: Disease management program | 5 (10.0) | 7 (13.2) | 0.612 | 40 |  |
| Personnel issues: Workplace organization / conditions | 2 (3.8) | 1 (1.9) | 0.614 | 41 |  |
| Cooperation with external medical colleagues: Diagnosis made by colleagues | 12 (24.0) | 15 (28.3) | 0.620 | 42 |  |
| Cooperation with external medical colleagues: Expertise / professional competence of colleagues | 11 (22.0) | 13 (25.0) | 0.721 | 43 |  |
| Cooperation with external medical colleagues: Treatment strategies of colleagues | 11 (22.0) | 10 (19.2) | 0.730 | 44 |  |
| Medicolegal investigation: Narcotic drug examination | 32 (72.7) | 38 (71.7) | 0.910 | 45 |  |
| Medicolegal investigation: Fire protection inspection | 22 (48.9) | 25 (59.0) | 0.990 | 46 |  |
| Cooperation with external medical colleagues: Agreements with colleagues | 17 (34.0) | 18 (34.0) | 0.997 | 47 |  |
| Cooperation with external medical colleagues: Treatment error of colleagues | 2 (3.9) | 3 (5.7) | 1.000 | 48 |  |
| Personnel issues: Work schedule | 2 (3.8) | 2 (3.7) | 1.000 | 48 |  |
| Personnel issues: Sick leave | 5 (9.6) | 5 (9.3) | 1.000 | 48 |  |
| Medicolegal investigation: Enforcement of penal proceedings | 0 (0) | 1 (1.9) | 1.000 | 48 |  |

* i = Rank; m = number of bivariate tests (65); Q = significance level of bivariate testing (0.05)

** (i/m)Q > p-value; i.e. p-values ≤0.001 are considered significant after correcting for multiple testing
